# Viewpoints of pedestrians with and without cognitive impairment on shared zones and zebra crossings

**DOI:** 10.1371/journal.pone.0203765

**Published:** 2018-09-11

**Authors:** Robyn Earl, Torbjorn Falkmer, Sonya Girdler, Susan L. Morris, Marita Falkmer

**Affiliations:** 1 School of Occupational Therapy, Social Work and Speech Pathology, Curtin University, Perth, Western Australia, Australia; 2 Rehabilitation Medicine, Department of Medicine and Health Sciences (IMH), Faculty of Health Sciences, Linköping University & Pain and Rehabilitation Centre, Linköping, Sweden; 3 School of Physiotherapy and Exercise Science, Faculty of Health Sciences, Curtin University, Perth, Western Australia, Australia; 4 School of Education and Communication, CHILD programme, Institute of Disability Research, Jönköping University, Jönköping, Sweden; Tokai University, JAPAN

## Abstract

**Background:**

Shared zones are characterised by an absence of traditional markers that segregate the road and footpath. Negotiation of a shared zone relies on an individual’s ability to perceive, assess and respond to environmental cues. This ability may be impacted by impairments in cognitive processing, which may lead to individuals experiencing increased anxiety when negotiating a shared zone.

**Method:**

Q method was used in order to identify and explore the viewpoints of pedestrians, with and without cognitive impairments as they pertain to shared zones.

**Results:**

Two viewpoints were revealed. Viewpoint one was defined by “confident users” while viewpoint two was defined by users who “know what [they] are doing but drivers might not”.

**Discussion:**

Overall, participants in the study would not avoid shared zones. Pedestrians with intellectual disability were, however, not well represented by either viewpoint, suggesting that shared zones may pose a potential barrier to participation for this group.

## Introduction

The built environment may act as either a barrier or facilitator to activity participation, and as such has an important influence on both physical and mental health [1–3]. An inclusive urban landscape should aim to promote engagement between users and reduce feelings of confusion, vulnerability and insecurity, particularly in those groups of people who are already at risk of social and civic disengagement [4–7]. In many cases when designing/redesigning an urban landscape it is those elements of the environment that create a physical barrier to participation (e.g., stairs) that are carefully considered and adapted to create an inclusive environment. However, there is rarely any consideration made for those differing or delayed cognitive processing ability, specifically autism spectrum disorder (ASD) and intellectual disability (ID).

There is an increasing focus nationally and internationally on the promotion of active travel [4–9]. An effective way to do this is to create urban spaces that encourage individuals to access their community on foot [4, 5, 10, 11]. However, by encouraging people out of their cars and onto the street there is the potential for increased risk of injury and crossing the road is one situation that pedestrians are at significantly increased risk of injury or death compared to drivers [9, 12]. Globally, an average of 270,000 pedestrians are killed each year, of these, most occur when the pedestrian is crossing the road [8]. Pedestrians with disabilities are even more at risk compared to their non-impaired peers [9, 12]. The major risk factors that impact on pedestrian safety are driver behaviour, pedestrian behaviour, road design and land use planning [9, 12]. There are a number of different types of road crossings that can be either signal controlled, as is often found at major intersections and on large motorways, or uncontrolled, that is, there is nothing to tell drivers when to stop and pedestrians when to go. The present study aimed to explore pedestrian viewpoints with regard to two types of uncontrolled, pedestrian priority traffic environments, namely, a black and white marked pedestrian crossing often referred to as a zebra crossing and an unmarked environment called a shared zone.

Shared zones are one urban design strategy that has become increasingly popular as a means of incorporating the needs of multiple modes of transport, while at the same time promoting social interaction between users [13, 14]. A shared zone is a “living street” that promotes equality between pedestrians, cyclists and motorists [13, 14]. Equality is fostered by the absence of traditional markings, gutters and kerbs that segregate roads from footpaths [13, 14]. Removal of traditional traffic management methods is believed to result in increased engagement between users and a slower more restrained flow of traffic, with a resultant traffic space that is safer and less congested than traditional traffic environments [13, 14]. Interactions within shared zones rely on informal social protocols and non-verbal communication strategies, such as eye contact and gestures [14]. While advocates of shared zones describe their benefits in terms of improvements in pedestrian safety, citing reduced injuries and fatalities post shared zone implementation[14], others have questioned whether the reduction in pedestrian crashes is actually achieved, at least in part, as a result of the creation of an environment that pedestrians perceive to be unsafe and intimidating and therefore avoid [15]. Successful negotiation of a shared zone relies, at least in part, on an individual’s ability to perceive, assess and immediately respond to social and environmental cues, skills that may be impacted by impairments in cognition and social processing. The safety and security of people with these impairments may therefore be differentially impacted when negotiating a shared zone.

Eye contact is an important form of nonverbal communication necessary in a shared zone to communicate intentions. Eye contact can be rapidly and independently identified within a complex scene, such as a shared zone, [16, 17] and can signal direction, intent and mental state [17]. Eye contact may play a role within a shared zone in allowing pedestrians and drivers to communicate their intent to one another, in order to avoid collision and maintain safety, particularly of pedestrians. In individuals with ASD gaze processing may be impaired at one or more levels [17–20]. An individual with ASD may have difficultly or fail to prioritise the eye contact of other individuals. Furthermore, they may have difficulty following and interpreting the gaze of another person (i.e., Theory of Mind) [17–19, 21]. They may also find it challenging to establish joint attention or co-ordinate their own focus on an object or event with another person [17–19, 21]. For people with ASD differences in patterns of eye contact in a shared zone may lead to missing or misinterpreting the gaze cues of other users, in particular drivers, or failing to accurately convey their own intentions when crossing traffic placing them at increased risk of collision. Users of shared zones with ASD may therefore find these environments challenging and experience increased anxiety, resulting in avoidance of these spaces [15]. While it could be suggested that pedestrians with ASD would have similar difficulties in all un-signalised traffic conditions, the relatively novel nature of shared zones and their increasing promotion and implementation in Western Australia and beyond warrants further research and focus. In order to better understand how shared zones impact on individuals, including those with ASD, it is vital to understand their views on these spaces.

Shared zones have high levels of environmental demand requiring an individual to quickly and accurately identify and respond to dynamic stimuli, tasks underpinned by cognitive processing skills. Pedestrians traversing a shared zone need to be able to identify and prioritise factors within the environment that pose a risk to their safety, namely motorised traffic, and respond accordingly. Individuals with ID experience impairments in planning, problem solving and interpreting their environment [19]. Within shared zones the informal rule structure and limited segregation between motorised and foot traffic [22] may result in individuals with ID failing to recognise that their safety is at risk until it is too late, resulting in injury or death. While there is limited previous research exploring the perceptions of people with physical disability [23] and visual impairment regarding shared zones [24] there is a distinct paucity of research exploring the viewpoints of individuals with and without social and cognitive impairments in regard to shared zones. As findings from previous research has suggested that those with physical and visual impairments tend to be more negative towards the concept and principles of shared zones than typical pedestrians [15, 23, 24] it is vital that a greater understanding the viewpoints of users with social and cognitive impairments is revealed. Identification of the viewpoints held by this population will allow greater insight into their ability to access their community and more specifically when using a shared zone.

In environments where pedestrians are able to choose between a shared zone and a zebra crossing it has been proposed that pedestrians will choose marked crosswalks to avoid what they perceive as an unsafe crossing point [15, 25]. In a single site study typical pedestrians were seen to divert from the more efficient line of travel that would have them traverse a shared zone in order to use a zebra crossing [15, 25]. While pedestrians may perceive marked cross walks to be safer, Zegeer, Esse (25) found the incidence of pedestrian crashes on marked cross walks to be higher than at unmarked crossing points in areas with an average daily traffic (ADT) amount greater than 10,000. In areas where ADT was less than 10,000 crash rates were comparable for both marked and unmarked crossing points, at approximately 0.25 pedestrian crashes per million pedestrian crossings [25]. Collectively, these findings suggest that pedestrians’ perceptions of safety may actually result in increased risk of injury [15, 25]. It is therefore important to reveal their viewpoints regarding shared zones, in order to understand how these perceptions may impact on pedestrian behaviour and overall safety. As such, the aim of this study was to reveal the viewpoints of individuals both with and without ID and ASD as they pertained to shared zones and zebra crossings, which has not been done before.

## Materials and methods

**Fig 1:**
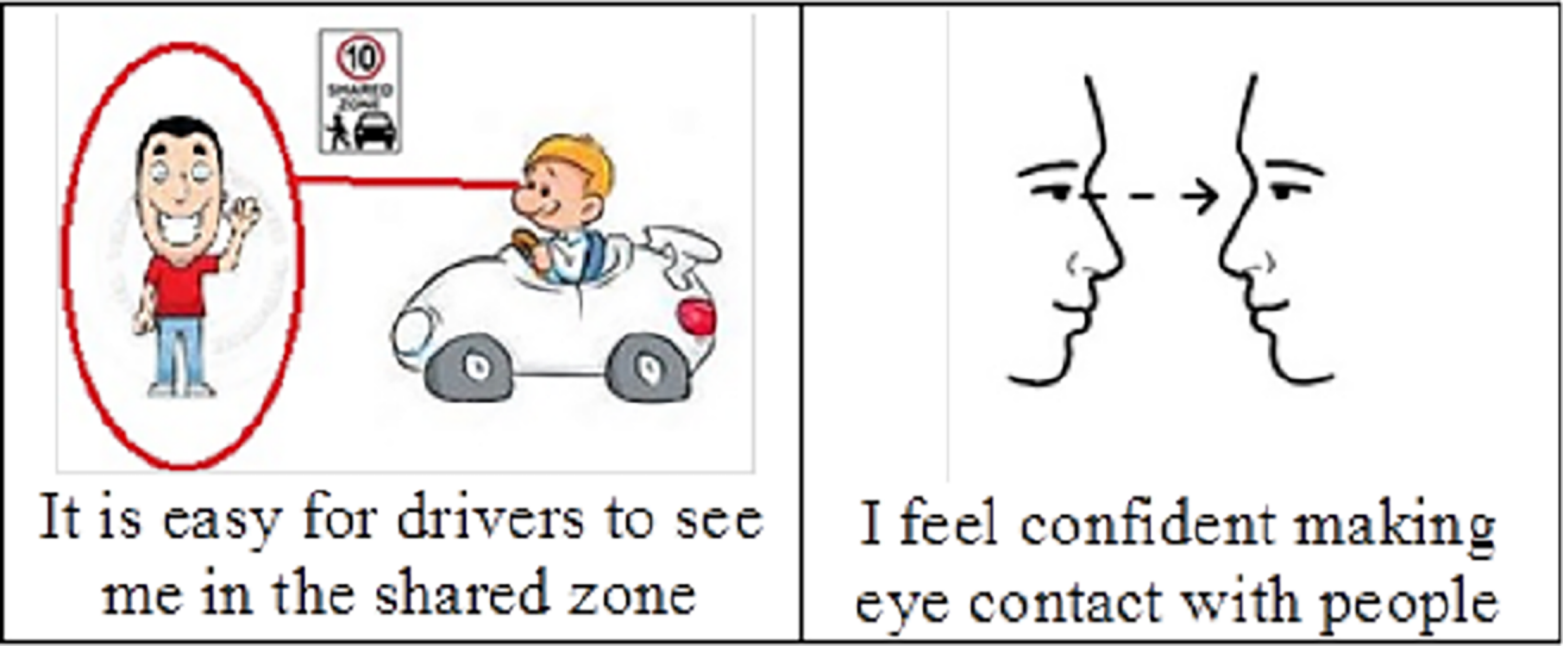
Example of statements presented with images to enhance understanding.

DesignQ method [26] was used in order to identify and explore the viewpoints of pedestrians, both with and without cognitive impairments. Q method utilises both inductive and deductive approaches, to explore an individual’s particular viewpoint on a subject, in this case as a pedestrian in a shared zone and zebra crossings [26]. The advantage of the Q method over traditionally interview methods is that it does not rely on participants’ verbal or written communication skills [26]. This meant that participants could be included in the study regardless of their verbal communication ability as long as they were able to comprehend basic spoken English. The Q method is built on statements that constitute a Q sort pack [26]. In this study these statements were paired with pictures, in order to enhance meaning and facilitate interpretation (Fig 1). All statements and pictures are presented in S1 Fig. Participants were also able to undertake the sort with the support of a carer or guardian enabling the use of terminology familiar to the participant, for example, the term “zebra crossing” may be described as a “marked pedestrian crossing” and linked to a familiar experience to them, e.g., the statement “I know the rules at a zebra crossing” can be described as “you know when we go to the football and we have to cross the road at the marked pedestrian crossing, do you know the rules that we have to follow to cross the road there?” Since the method forces the participant to sort all statements across the grid, a constant comparison approach was used by the researchers together with the carers/guardians in which any lack of understanding was discovered and the meaning of the statements clarified. Indeed Q method has been proven to be an effective tool for exploring the viewpoints of these target groups in previous research [27–31]

Participants: A total of 62 participants, from three groups, completed the Q sort. Group one consisted of 21 typically developing (TD) adults who had no known diagnosis of ASD, ID or physical impairment. Group two comprised of 21 adults who had a diagnosis of mild to moderate ID as reported by a parent, guardian or carer. Group three comprised of 20 adults who met the Diagnostic and Statistical Manual of Mental Disorders, Fourth Edition (DSM-IV) criteria of high-functioning autism or Asperger’s Syndrome. All participants were living in Western Australia, and were independently mobile in that they did not use a walker, were in a wheel chair or used any other assistive device to negotiate a shared zone or zebra crossing. The research project was approved the Curtin Human Research Ethics committee (ref # HR59/2014) and was conducted according to the principles expressed in the Declaration of Helsinki. Participants provided voluntary informed consent and where participants were unable to give informed consent, a legally authorised representative provided voluntary written consent and the participant provided assent.

Q sort studies aim to select participants that enable the description of variation between known groups. Table 1 outlines the demographics and characteristics of all groups. Participants were recruited from across Western Australia through specialist student mentoring programs, disability service providers, radio advertising and social media.

**Table 1:** Participant demographic data and driving status.

	TD (n = 21)	ID (n = 21)	ASD (n = 20)	All (n = 62)
Age (years)				
Mean (SD)	38.67 (20)	35.95 (13.3)^a^	25.50 (12.3)	33.46 (16.4)
Gender (n)				
Male	7 (33%)	14 (33%)	17 (85%)	38 (61%)
Female	14 (67%)	7 (67%)	3 (15%)	24 (39%)
Driving Status (n)				
Driver	20 (95%)	5 (24%)	14 (70%)	39 (63%)
Non-Driver	1 (5%)	16 (76%)	6 (30%)	23 (37%)

Percentages displayed are within group percentages unless otherwise stated. TD = typically developed, ID = Intellectual disability, ASD = Autism Spectrum disorder.

a One participant in the ID group did not report their age.

### Tools

**Fig 2:**
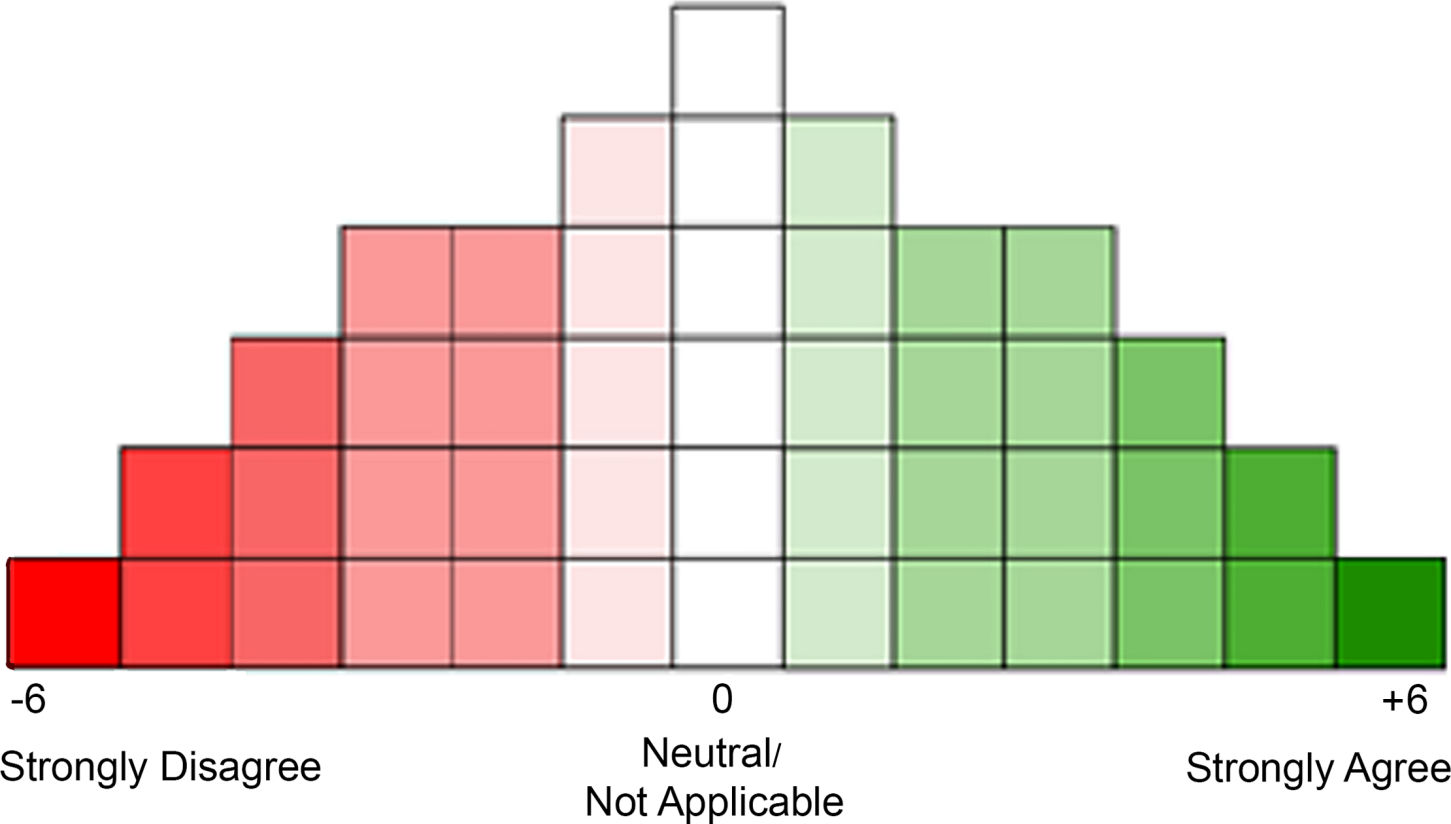
Q sort grid.

A Q sort, which consists of a Q sort pack (a set of predetermined statements) and a corresponding normally distributed grid (see Fig 2) was developed specifically to meet the needs of this study. For this study the Q sort pack consisted of 44 statements with images and a normally distributed grid of 44 squares. The development of the statements and the size of the grid is outlined below.

#### Proceduresdeveloping the concourse

A set of statements exploring possible viewpoints on the pedestrian experience while negotiating a shared zone and zebra crossing was developed based on findings from a review of the available literature [14, 32–34]. The statements were then reviewed and revised in consultation with experts in the field of traffic safety, ID and ASD. Initially, 56 statements were selected and piloted using two adults with ASD, one adult with ID and their carer. The images (Fig 1) were also reviewed and refined in collaboration with these adults, as well as with experts in the field of intellectual disability and ASD. Pilot participants were asked if they believed the statements accurately reflected their experience as a pedestrian in a shared zone or on a zebra crossing, and if they felt that anything was missing and if so what could be added. Following piloting and refinement, a total of 44 statements were selected and comprised the final Q sort pack (S2 Appendix A).

#### Administering the Q sort

**Fig 3:**
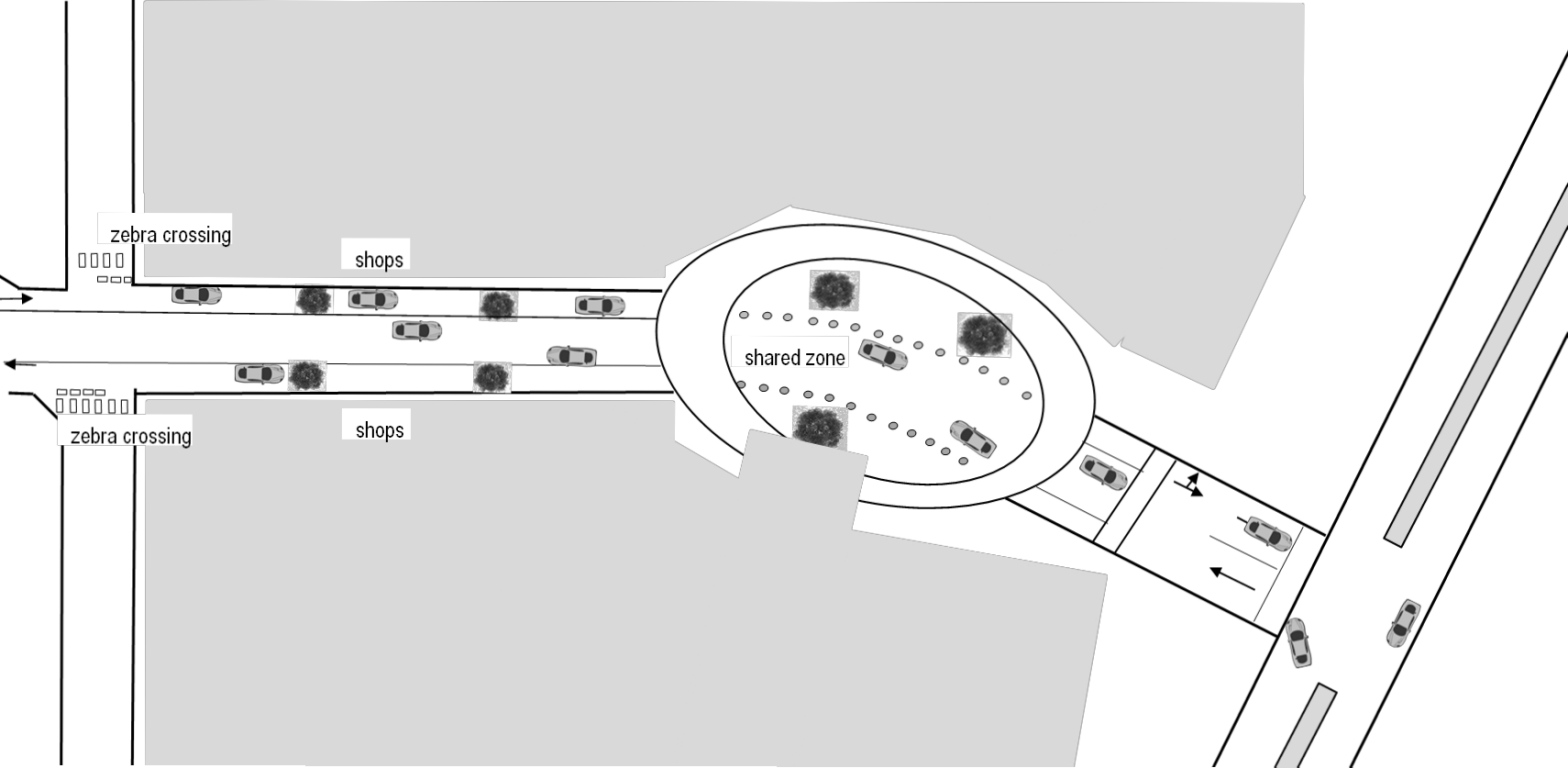
The shared zone/ road runs through the centre of a shopping mall in two directions. It is clearly marked as a reduced speed zone (<10km/h) and pedestrians have right of way. It is a small area that is designated for crossing that has a moderate amount of foot and motorized traffic. The road is marked with bollards and is very closely locate to multiple zebra crossings, acted as the control crossing area.

Participants were met at a designated shared zone at a local shopping centre in Perth, Western Australia (Fig 3). Participants were instructed to traverse the shared zone and to cross a nearby zebra crossing during simulated shopping tasks. All participants had at least one exposure to the shared zone and zebra crossing prior to completion of the Q sort. In completing the Q sort participants were asked to reflect on their time in the shared zone and on the zebra crossing, as well as on any previous experiences that they had as a pedestrian.

Participants were instructed to carefully read all 44 statements and to consider the extent to which they agreed or disagreed with each of the statements in relation to their experience as a pedestrian. Participants sorted the statements (Fig 1) onto a normally distributed sorting grid (Fig 2) with a continuum of “strongly disagree” to “strongly agree” with ranking values of -6 (strongly disagree) to 0 (neutral/not applicable) to +6 (strongly agree). The sorting grid prescribed the maximum number of statements for each rank allowing for one statement per square as indicated in Fig 2. For the sort to be complete each square needed to be filled. It was also made clear to participants that there were no right or wrong answers, and that statements could be rearranged until they were satisfied that the sort was a true representation of their views. Participants took an average of 30 minutes (SD = 18.7) to complete the sort.

### Data analysis

#### Factor analysis

The PQmethod software package [35] was used to analyse the Q sorts from each participant (n = 62). The PQmethod package allows for the main viewpoints, referred to as factors, to be extracted which are then analysed using by person varimax rotation factor analysis. This analysis method positions the factors such that the overall rotated solution best accounts for and reflects the variance explained; the results in each individual Q sort loading on a single factor, thus revealing the primary viewpoint of that person (Table 2). The PQmethod software detects those individual Qsorts which exemplify a particular viewpoint as well as those participants who arrange their statements in a similar manner. Statements that do not significantly differ across viewpoints (consensus statements) are also identified (Table 2).

**Table 2:** Factor loadings of individual sorts.

Characteristics in respect to gender, age, diagnostic group, and driver status				Factor	
				1	2
Male	20	ASD	Driver	**0.6265**	0.4544
Male	20	ASD	Driver	**0.5258**	0.077
Male	23	ASD	Driver	**0.7397**	0.3496
Male	18	ASD	Driver	**0.6852**	0.3891
Male	18	ASD	Driver	**0.7725**	0.3104
Male	18	ASD	Non-driver	**0.689**	0.4939
Female	19	ASD	Driver	**0.3624**	0.1234
Male	19	ASD	Non-driver	**0.7969**	0.3129
Female	39	ASD	Driver	**0.5737**	0.1483
Male	24	ASD	Driver	**0.7255**	0.0698
Male	20	ASD	Non-driver	**0.7025**	0.2783
Female	18	ASD	Non-driver	**0.7146**	0.2121
Male	20	ASD	Driver	**0.7596**	0.4952
Male	19	ASD	Driver	**- 0.6748**	-0.3589
Male	64	ASD	Driver	**0.3766**	0.3376
Male	23	ID	Driver	**0.6032**	0.2575
Female	37	ID	Non-driver	**0.4258**	0.0907
Female	40	ID	Non-driver	**0.3298**	-0.1363
Male	30	ID	Non-driver	**0.3109**	0.2876
Female	22	ID	Non-driver	**0.433**	0.2251
Male	51	ID	Driver	**0.6239**	-0.064
Female	19	TD	Driver	**0.7327**	0.386
Female	26	TD	Driver	**0.3621**	0.1477
Female	72	TD	Non-driver	**0.5516**	0.0777
Female	21	TD	Driver	**0.6358**	0.3618
Female	61	TD	Driver	**0.5188**	0.1488
Male	20	TD	Driver	**0.7809**	-0.1872
Female	31	TD	Driver	**0.775**	-0.1041
Male	71	TD	Driver	**0.6382**	0.3683
Male	32	TD	Driver	**0.5548**	0.5113
Male	21	TD	Driver	**0.8483**	-0.0324
Female	22	TD	Driver	**0.6381**	0.3076
Female	21	TD	Driver	**0.6722**	0.0704
Male	61	TD	Driver	**0.4748**	-0.1924
Male	30	TD	Driver	**0.8622**	0.096
Female	22	TD	Driver	**0.7179**	-0.0944
Female	57	TD	Driver	**0.5411**	0.3049
Female	26	TD	Driver	**0.6248**	0.2701
Female	47	TD	Driver	**0.5797**	0.3967
Male	19	ASD	Driver	0.2663	**0.5327**
Male	28	ASD	Non-driver	0.3963	**0.5393**
Male	18	ASD	Non-driver	-0.25	**0.5175**
Male	43	ASD	Driver	0.2771	**0.5225**
Male	20	ID	Non-driver	0.1416	**0.5895**
Male	29	ID	Non-driver	0.399	**0.5922**
Female	48	ID	Non-driver	0.4974	**0.6169**
Male	23	ID	Non-driver	0.0467	**0.3505**
Female	48	ID	Driver	0.2387	**0.6396**
Male	33	ID	Non-driver	0.1482	**0.5182**
Female	22	TD	Driver	0.3616	**0.5899**
Female	62	TD	Driver	0.3686	**0.5985**
Male	43	ASD	Driver	- 0.2567	0.2735
Male	46	ID	Non-driver	0.2165	0.2811
Male	28	ID	Non-driver	0.2255	-0.1526
Male	19	ID	Driver	0.2919	0.0593
Male		ID	Non-driver	- 0.0145	0.1364
Male	64	ID	Non-driver	0.0093	0.2267
Female	44	ID	Non-driver	0.0209	-0.188
Male	57	ID	Driver	- 0.0071	0.1051
Female	22	ID	Non-driver	- 0.0772	0.2656
Male	35	ID	Non-driver	0.2216	-0.087
Male	68	TD	Driver	0.1828	0.0946
Explained Variance (%)				27	12
Number of defining sorts				39	12
Factor score correlation					0.6072

Numbers in bold indicate defining sort.

TD = Typically Developed, ASD = Autism Spectrum Disorder, ID = Intellectual Disability

**Fig 4:**
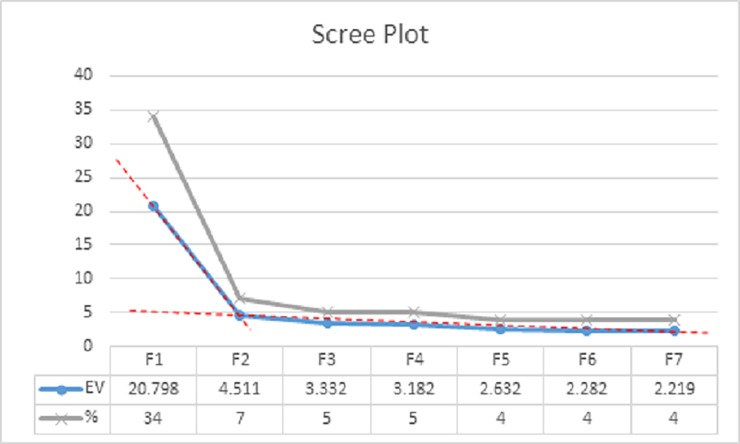
Scree plot dotted line assists to identify the inflection point. EV = eigenvalue. % = Percentage of explained variance.

Application of a step-wise hierarchy of criteria revealed the main viewpoints, i.e., factors. The first criteria, known as the “magic number seven”, requires the extraction of seven factors, the default number defined in PQmethod [35] and was the starting point for this analysis. Secondly, the Kaiser-Guttman criterion was used applied to the eigenvalues of extracted factors. According to this criterion only those factors with an eigenvalue greater than 1.00 should be considered for inclusion [36]. In this study all seven of the extracted factors met this criterion. The third criterion was the acceptance of interpretable factors which had two or more significantly loaded sorts after extraction. Significant loading is calculated by the equation 2.58 x (1/√(number of statements in Q set)), and in this case 2.58 x 1/ (√44) = ) = 0.39 (rounded to 2 decimal places) [26]. Only those sorts that loaded on a single factor were included, with those that loaded on more than one factor considered to be confounding and thus excluded. Factors 1, 2, 3, 4 and 6 all met this criteria. Next, Humphrey’s Rule was applied to the remaining factors. Humphrey’s Rule states that a factor is only regarded as significant if multiplication of the two highest absolute loadings is greater than twice the standard error [26]. Standard error for this study was defined as 0.15 and only factors 1, 2 and 3 met this criterion. Finally, the “scree test” was applied to all seven factors (Fig 4). This test involved plotting each factor’s eigenvalue against the number of factors. The final set of factors is determined by the point at which the gradient of the curve changes. The scree test indicated that 2 factors should be extracted and the final varimax rotation and analysis was run with only the first two factors. Ultimately, the determination of the final set of factors should account for a significant portion of the variance, typically anything in excess of 30–40% is acceptable [26]. Factors in the present study were extracted using Centroid Factor Extraction Method and accounted for 39% of the variance. Once the number of factors was identified, researchers in the areas of ASD, ID and transportation were invited to interpret the findings. Agreement on the title of each factor was reached via group discussion and consensus, a step crucial in limiting researcher bias [26].

### Ethical considerations

Informed consent was obtained from all participants prior to completion of the Q sort. Guardian consent was also obtained where deemed appropriate. All data were de-identified ensuring confidentiality. The study and its procedures were approved by Curtin University Human Research Ethics Committee in Western Australia (HR-56/2014) and also conformed to the Declaration of Helsinki [37].

## Results

### Interpretation of factors

Two viewpoints emerged following analysis of the participants Q sorts. The factors were defined by 51 of the participants (82%) as 11 participants did not load significantly on either factor. Table 2 shows the Q sorts and the individual characteristics of those participants who loaded significantly on each factor.

### Factor interpretation

#### Viewpoint 1: “Confident Users [in both the shared zone and on the zebra crossing]”

Viewpoint 1 was defined by 39 participants. These participants came from all three groups, 18 (46%) were TD adults, 15 (39%) were adults with ASD and 6 (15%) were adults with ID. Table 3 shows the demographic and driving status by group for those participants that loaded on viewpoint 1.

**Table 3:** Demographic data for participants that loaded on factor 1.

	TD (n = 18)	ID (n = 6)	ASD (n = 15)	All (n = 39)
Age (years)				
Mean (SD)	36.67 (19.1)	33.83 (11.1)	23.93 (12.3)	31.33 (16.5)
Gender (n)				
Male	6 (33%)	3 (50%)	12 (80%)	21 (54%)
Female	12 (67%)	3 (50%)	3 (20%)	18 (46%)
Driving Status (n)				
Driver	17 (94%)	2 (33%)	11 (77%)	30 (77%)
Non-Driver	1 (6%)	4 (67%)	4 (23%)	9 (23%)

Percentages displayed are within group percentages unless otherwise stated. TD = Typically Developed, ASD = Autism Spectrum Disorder, ID = Intellectual Disability

Theses participants felt confident crossing the road at a controlled crossing (statement 35 in S1 Table:+6,). They also felt confident (24:+5) and secure (41:+4) crossing the road at a zebra crossing and confident in the shared zone (23:+5). They did not feel they needed someone with them at the zebra crossing (26:-5) or in the shared zone (40:-6). They also did not avoid shared zones (29:-4) or zebra crossings (18:-4) when accessing their community. These participants also did not perceive zebra crossings as dangerous (42:-5) or that the rules of a zebra crossing were hard to understand (27:-4), knowing when (39:+4) and where (41:+4) to cross the road at a zebra crossing, as shown in Table 4.

**Table 4:** Viewpoint 1: Confident users.

		Viewpoint	
No.	Statement	1	2
35	I feel confident crossing the road at traffic lights	+6	+3
23	I feel confident crossing the shared zone	+5	+3
24	I feel confident crossing the zebra crossing	+5	+4
38	I feel secure using a zebra crossing	+4	+1
39	I know when to cross a zebra crossing	+4	+5
41	I know where to cross the road at a zebra crossing	+4	+5
18	I stay away from zebra crossing	-4	-3
27	The road rules of the zebra crossing are hard to understand	-4	-2
29	I stay away from shared zone	-4	-4
42	Zebra crossing are dangerous	-5	0
26	I need someone with me when I cross a zebra crossing	-5	-5
40	I need someone with me in the shared zone	-6	-6

Note: All 44 statements are presented in S1 Table.

#### Viewpoint 2: “I know what to do [in both the shared zone and on the zebra crossing] but drivers might not [follow the rules]”

Viewpoint 2 was defined by 12 participants. As with viewpoint 1 all groups were represented (Table 5). These participants was defined by two typically developing adults (17%), 4 adults with ASD (33%) and 6 adults with intellectual impairment (50%).

**Table 5:** Viewpoint 1: Confident users.

		Viewpoint	
No.	Statement	1	2
**35**	I feel confident crossing the road at traffic lights	+6	+3
**23**	I feel confident crossing the shared zone	+5	+3
**24**	I feel confident crossing the zebra crossing	+5	+4
**38**	I feel secure using a zebra crossing	+4	+1
**39**	I know when to cross a zebra crossing	+4	+5
**41**	I know where to cross the road at a zebra crossing	+4	+5
**18**	I stay away from zebra crossing	-4	-3
**27**	The road rules of the zebra crossing are hard to understand	-4	-2
**29**	I stay away from shared zone	-4	-4
**42**	Zebra crossing are dangerous	-5	0
**26**	I need someone with me when I cross a zebra crossing	-5	-5
**40**	I need someone with me in the shared zone	-6	-6

Note: All 44 statements are presented in S1 Table

They felt that it was important that they could cross a zebra crossing independently (31:+6). Similar to the participants sharing viewpoint 1, they also knew where (41:+5) and when (39:+5) to cross the zebra crossing. Also, as with those of viewpoint 1, these users did not need someone with them when they crossed a zebra crossing (26:-5) or were in the shared zone (40:-6). The “who know what to do” participants were wary of drivers following the rules (24:-4) and stopping to let them cross at a zebra crossing (30:-4) or stopping to allow them to cross the shared zone (2:-5). They also believed that the road and footpath should be separate (7:+4), as shown in Table 6.

**Table 6:** Viewpoint 2: “I know what I’m doing but drivers might not”.

		Viewpoint	
No.	Statement	1	2
**31**	It is important I can walk by myself across a zebra crossing	+3	+6
**41**	I know where to cross the road at a zebra crossing	+4	+5
**39**	I know when to cross a zebra crossing	+4	+5
**7**	The road and the footpath should be separate	0	+4
**12**	I know when to cross a shared zone	+3	+4
**24**	I feel confident crossing the zebra crossing	+5	+4
**28**	Drivers follow the rules of a zebra crossing	0	-4
**30**	I know when a car is going to stop and let me cross the zebra crossing	+2	-4
**29**	I stay away from shared zone	-4	-4
**2**	Cars always stop to let me cross the shared zone	-1	-5
**26**	I need someone with me when I cross a zebra crossing	-5	-5
**40**	I need someone with me in the shared zone	-6	-6

Note: All 44 statements are presented in S1 Table

### Consensus statements

There were 17 consensus statements (indicated with a,b in S1 Table) with no statistically significant difference in the scores across both viewpoints (S1 Table). They shared significant negative rankings for statements 40 (-6), 26 (-5) and 29 (-4) suggesting that participants sharing both viewpoints were confident in accessing their community independently and were unlikely to avoid a shared zone in the future. They were also confident crossing a zebra crossing (24:+4, +5) and being in the shared zone (23:+5, +3). They all knew when (39:+4, +5) to cross a zebra crossing and shared zone (12:+3, +4) and where (41:+4, +5) to cross a zebra crossing. It was agreed across these participants that being able to walk across a shared zone independently was moderately important (25:+3). Statement 4 (“Bollards get in my way”) was ranked negatively across both viewpoints (-3) suggesting that neither viewpoint found bollards to be a barrier to mobility. Statements 6 and 14 were ranked moderately positive (+1 to +3) indicating that participants across both viewpoints knew where to cross the shared zone but they may have difficulty seeing oncoming cars in the shared zone, which, in turn, may have affected their sense of security in the shared zone (20:+2). This is also reflected in the negative ranking (-1) of item 43, “more shared zones would make it easier for me”. Participants across both viewpoints were moderately confident making eye contact with other people (19:+2). They all gave a neutral ranking (0 to +1) in regards to using the noise signal to cross at traffic lights (item 5) and using signs to stay safe from cars (15:0).

## Discussion

The fact that only two viewpoints were revealed and the high levels of similarity between them is an interesting finding in itself. Typically, Q sorts reveal three or more diverse viewpoints [30, 31], however in this study there was a homogeneity in the pedestrians’ viewpoints. Specifically, the main similarity between the two viewpoints is that the bulk of the defining statements were around zebra crossings in preference to shared zones. This may be due to the increased familiarity and past exposure to zebra crossings. Furthermore, the homogeneity between the viewpoints may also suggest, as suggested by previous research, that any traffic calming measures, including shared zones that slow vehicle speeds are good for all users [22, 38–40]. Hence, this may account for the finding that those who loaded on both viewpoints would not avoid the shared zone or deem it unsafe. While there was similarity across viewpoints, the most notable difference between the two appeared to be that those sharing viewpoint two did not trust drivers to abide by traffic rules, either at a zebra crossing or within the shared zone. This difference can be explained in part by exploring the demographic characteristics of the participants sharing the different viewpoints.

Viewpoint one reflects a group that was confident in themselves and in their ability to know when and where to cross the road, particularly at a zebra crossing. Those pedestrians that loaded on viewpoint one did not rank statements reflecting the behaviour and perceived intentions of drivers significantly, suggesting that their ability to interpret driver behaviour did not impact upon their decision of when and where to cross the road. This may have been because this group was primarily comprised of drivers and as such were more familiar with the behaviour and movements of other drivers. While there is limited evidence suggesting that there is any difference in the skills of driver and non-driver pedestrians with regards to road crossing behaviour [41–43], the added skills held by drivers when crossing the road may be reflective of their confidence when traversing traffic, particulary at the zebra crossing. The limitation of this study as with previous research was that it did not explore the connection between the pedestrians’ intention to cross and their actual behaviour which may differ [44]. Clearly, further research examining the crossing behaviour of drivers versus non-drivers is needed to better understand the relationship between a person’s driving status and their behaviour as a pedestrian.

On average participants in the confident group were younger, a fact which may partially explain their confidence and why they did not rank the actions of drivers in a way that indicated they played a significant role in their decision to cross. A large body of evidence supports the idea that younger adults are more confident and more likely to take risks than their older counter parts [1, 41, 45–47]. A surprising finding was that participants with ASD loaded on the first view point as much as those without any impairment. As raised in the introduction, current literature suggests that the core impairments of ASD, particuarly in the area of social processing may lead those with ASD to experience difficulty in road crossing at uncontrolled crossing points such as a shared zone, and to a lesser extent a zebra crossing, where personal safety is maintained, primarily through engaging in social interaction with drivers [17–20]. It could be assumed that the impairments associated with ASD would lead to increased anxiety and insecurity when crossing the shared zone, this however does not appear to have been the case in this study. It may be because social interaction, specifically eye contact, was not the primary strategy employed by these pedestrians with ASD when traversing the shared zone [48]. Further research is needed to understand how people with ASD visually scan and attend to traffic relevant information, as while they were as confident as those without impairment they may not have been as equally competent when crossing.

While the participants with ASD appeared to be as confident as their typically developing peers, participants with ID were less confident, loading less frequently than the TD participants and participants with ASD on viewpoint one. This finding was expected, and is consistent with previous research finding pedestrians with ID were less adept at interpreting social stimuli than those without ID [32], and less confident when accessing their community [19, 49, 50]. A lack of knowledge has also been identified as a barrier to social inclusion for people with ID [49], however, those that shared viewpoint two were confident in their own knowledge of the road rules at the zebra crossing and in the shared zone. They were instead wary of drivers being knowledgeable of and following the rules in either the shared zone or on the zebra crossing. The wariness towards other road users, particularly drivers, may lead to pedestrians with ID to be more cautious and adopt safer road crossing behaviours, but may also lead to them experiencing heightened anxiety when accessing the community [15, 23, 24, 49]. This may have many negative outcomes for road safety, including the avoidance of certain crossing situations, such as shared zones, and funnel vulnerable users towards alternate crossing points, such as a zebra crossing, which under some circumstance may not be safer and at times present more risk [15, 25, 51, 52]. Increased anxiety may also contribute to decreased community participation [2, 53, 54], negatively impacting on health and well-being, compounding the cost of disability to individuals and society [55–57].

Overall, neither viewpoint suggested that participants would avoid a shared zone in the future. However, there were a greater number of participants with ID, compared to other pedestrians, which were not represented by either viewpoint (n[TD] = 1, n[ASD] = 1, n[ID] = 9). It is possible that these participants do have difficultly in the shared zone and have views that reflect this but there was not enough similarity in the unloaded sorts to meet the criteria for a third viewpoint. Further research with a larger number of participants may result in a third factor that represents the viewpoints of these participants being revealed. As such, it is also crucial that future research explores how pedestrians, including and specifically those with ID, visually scan and attend to the environment when crossing a zebra crossing or when in a shared zone in order to better understand why they are less confident and how they interact with other road users, namely drivers.

## Limitations

The primary limitation of this study was the reliance on self-report in regards to diagnosis and the degree of impairment of participants. Due to the nature of this study it was not practically feasible or ethical to assess each individuals’ level of impairment. The interactions between IQ, social skills and views towards the shared zone and zebra crossing cannot be explored and should be the focus of future study. This would help to better identify for which group/s, if any, shared zones present a barrier to participation in society. It may also help to identify those more likely to make risky/unsafe decisions when crossing the road at either a zebra crossing or in a shared zone.

Secondly, this study was conducted in a small shared zone in Perth, Western Australia, where shared zones are a relatively new and novel concept which many of the participants had not heard of nor experienced, prior to the trial. Future studies exploring the viewpoints of those persons both with and without impairment in areas with longer established shared zones may yield different results based on the users’ increased experience.

Thirdly, whilst the gender and age balances of the group were different, all three groups were representative samples with regard to age and gender for the user groups of the Shared Zone [48]. The analysis is based on this presumption.

Finally, the participants in this study were primarily young adults. As there is a known connection between age and road crossing intention and behaviour, further research exploring the viewpoints of an older cohort is required to gain a deeper understanding of the effect shared zones and zebra crossings have on them. It would allow for a better understanding of how the wider population perceives these areas and the implication these may have on pedestrian travel patterns and behaviours, particularly as they pertain to safety.

## Conclusions

Shared zones are a unique traffic management solution, which is reported to reduce traffic speed while maintaining traffic flow, increasing driver attentiveness and improving safety for all road users14. This study sought to reveal the viewpoints of pedestrians with regards to shared zones. It was found that shared zones were not regarded by the majority of pedestrians with and without cognitive impairment to be a barrier to community participation. Pedestrians in this study would not avoid shared zones and did not perceive them to be dangerous. A number of concerns regarding driver behaviour were exposed through viewpoint two, namely obeying the rules and stopping for pedestrians. Future research and planning should consider how best to address these concerns and manage driver behaviour. Furthermore, a number of pedestrians with ID did not load on either viewpoint suggesting that this is a group that may find shared zones to be a challenging and confronting environment. These results were not conclusive however, and further research exploring how people with ID navigate and cognitively process the shared zone environment would be beneficial. Consequently, the implementation of a shared zone should be assessed on a case-by-case basis and should not be made unless they actually offer pedestrians what they perceive to be a safe urban environment accessible for all, so as not to deter foot traffic and create a potential perceived barrier to community participation.

## Supporting information

S1 Fig The 44 Q sort statements used in the study. (DOCX)

S1 Table Q-set statements, factor arrays of viewpoints (Q rank) and z-scores. Note. ^ Indicates distinguishing statement p<0.05. * Indicates distinguishing statements p < 0.01. a Indicates consensus statement non-significant at p> 0.01. b Indicates consensus statements non-significant at p > 0.05. N.B. there were no distinguishing statements that met significance either at p<0.05 or p<0.01 for factor 2.
(DOCX)
