# Supplementary material for: Viewpoints of pedestrians with and without cognitive impairment on shared zones and zebra crossings
Source: PLoS One. 2018 Sep 11;13(9):e0203765. doi: 10.1371/journal.pone.0203765 (PMC6133379; doi:10.1371/journal.pone.0203765)
Supplement: S1 Fig — (DOCX) [file pone.0203765.s001.docx]

| 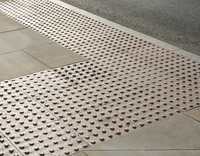  I use the tactile paving | [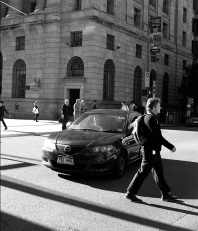](http://brisstreet.files.wordpress.com/2011/08/shared-zone-george-st-sdim1452_edited-1.jpg)  Cars always stop to let me cross the shared zone | 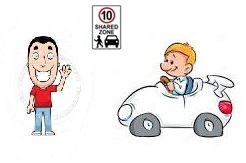It is easy to know when a car is going to stop to let me cross the shared zone |
| --- | --- | --- |
| 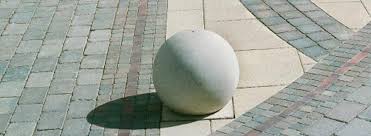  Bollards get in my way | [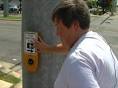](https://www.google.com.au/url?q=http://guidedogmanual.weebly.com/shelley-in-the-news.html&sa=U&ei=FttdU4OiK8TQkwW27YCQBQ&ved=0CD4Q9QEwBw&usg=AFQjCNEJNJuq916TWJzbW2EFXVLZB5nkAA)  I use the beep at traffic lights to know when to cross the road | 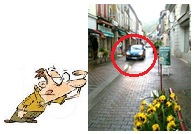  It is easy to see oncoming cars in a shared zone |
| [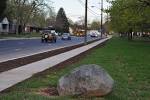](https://www.google.com.au/url?q=http://www.urbanindy.com/2011/04/13/new-sidewalks-in-midtown-indy/&sa=U&ei=HtldU_mWBtHakgXEl4HwCA&ved=0CDAQ9QEwAQ&usg=AFQjCNEMWj0GzBZ_yfN2BYTdemjpW654TA)  The road and footpath should always be separate | 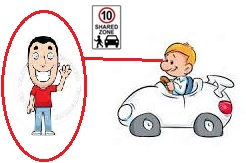  It is easy for drivers to see me in the shared zone | 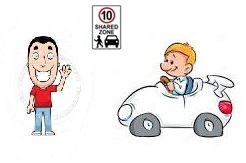  Drivers always follow the road rules in a shared zone |
| 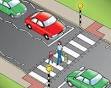  More zebra crossings would make it easier for me | Cars feel too close to me at the zebra crossing 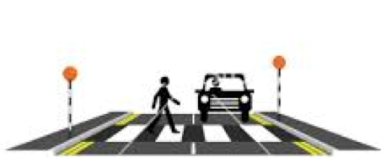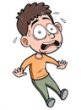 | [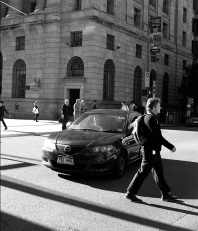](http://brisstreet.files.wordpress.com/2011/08/shared-zone-george-st-sdim1452_edited-1.jpg)  I know **when** to cross a shared zone |
| 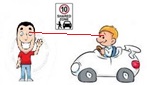  Drivers make eye contact with me in the shared zone | [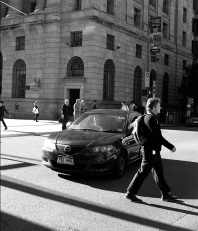](http://brisstreet.files.wordpress.com/2011/08/shared-zone-george-st-sdim1452_edited-1.jpg)  It is easy to know **where** to cross a shared zone | 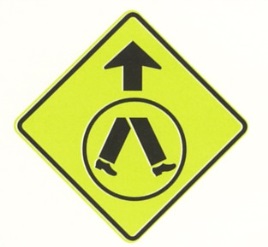  Signs help me to stay safe from cars |
| 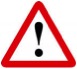  Shared zones are dangerous | 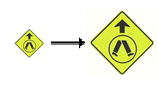Signs in the shared zone need to be bigger | 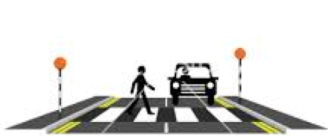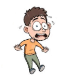 I stay away from zebra crossings |
| 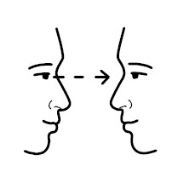  I feel confident making eye contact with people | 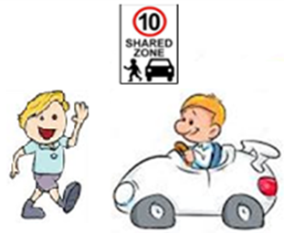  I feel **secure** being in a shared zone | [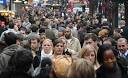](https://www.google.com.au/url?q=http://www.dailymail.co.uk/news/article-1222049/Crowded-Britain-heading-70m-migration-causes-population-rise-faster-before.html&sa=U&ei=YtldU_2vF8mllAXAq4HwCQ&ved=0CDIQ9QEwAg&usg=AFQjCNGfRLC7KxpB0RMokNH7smxLipXc5A)  I like it when there are lots of people around |
| [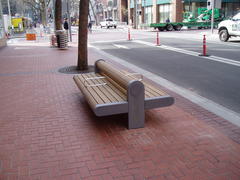](http://blog.oregonlive.com/oldtown/2009/06/Street%20Furniture.JPG)  I like to have somewhere to sit down when I go out | 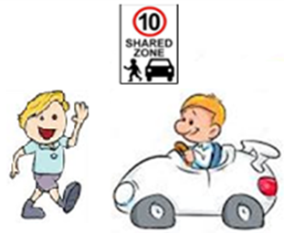  I feel **confident** crossing a shared zone | [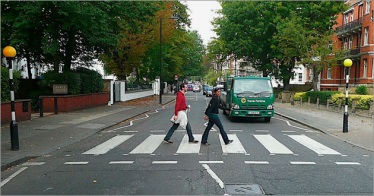](http://www.wired.com/images_blogs/thisdayintech/2011/10/zebra_crossing_630px.jpeg)  I feel confident crossing the road at a zebra crossing |
| 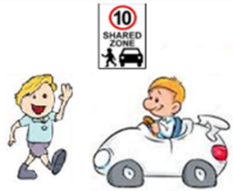  It is important that I can walk by myself across a shared zone | 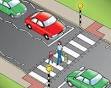  I **need** someone with me when I am at a zebra crossing | 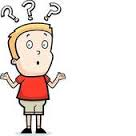The road rules at a zebra crossing are hard to understand |
| 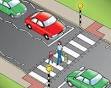  Drivers always follow the rules of a zebra crossing | I stay away from shared zones 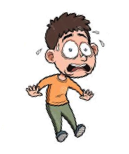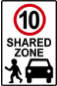 | 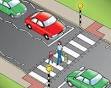  It is easy to know when a car is going to stop and let me cross a zebra crossing |
| 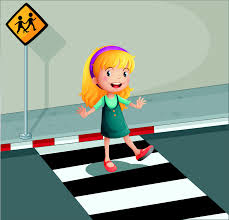  It is important that I can walk by myself across a zebra crossing | 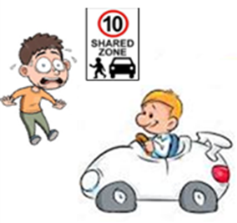  Cars feel too close to me in the shared zone | 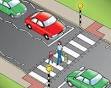  Cars always stop to let me cross the road at a zebra crossing |
| 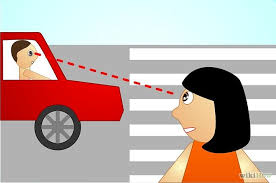Drivers make eye contact with me at a zebra crossing | [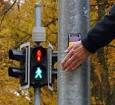](https://www.google.com.au/url?q=http://www.abc.net.au/science/articles/2010/05/04/2889699.htm&sa=U&ei=wtldU8G8Lca5lQWF_ICoDQ&ved=0CDAQ9QEwAQ&usg=AFQjCNFlnXmJ4UsNDQuMqk_UInOPh4sByg)  I feel confident crossing the road at traffic lights | 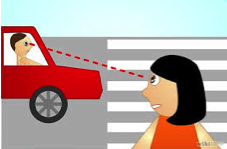 It is easy to see oncoming cars at the zebra crossing |
| 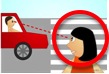  It is easy for drivers to see me at a zebra crossing | 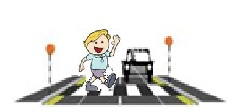 I feel secure using a zebra crossing | 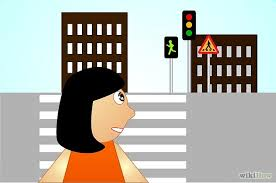  I know **when** to cross a zebra crossing |
| 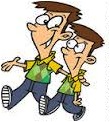  I **need** someone with me when I am in a shared zone | 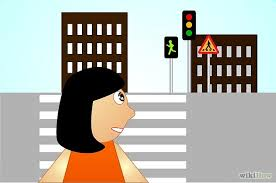  It is easy to know **where** to cross the road at a zebra crossing | 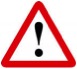  Zebra crossings are dangerous |
| 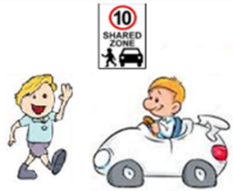  More shared zones would make it easier for me | 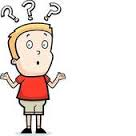The road rules of the shared zone are hard to understand |  |
|  |  |  |
