# Supplementary material for: Viewpoints of pedestrians with and without cognitive impairment on shared zones and zebra crossings
Source: PLoS One. 2018 Sep 11;13(9):e0203765. doi: 10.1371/journal.pone.0203765 (PMC6133379; doi:10.1371/journal.pone.0203765)
Supplement: S1 Table — Note. ^ Indicates distinguishing statement p<0.05. * Indicates distinguishing statements p < 0.01. a Indicates consensus statement non-significant at p> 0.01. b Indicates consensus statements non-significant at p > 0.05. N.B. there were no distinguishing statements that met significance either at p<0.05 or p<0.01 for factor 2. (DOCX) [file pone.0203765.s002.docx]

|  |  | View Point | | | |
| --- | --- | --- | --- | --- | --- |
|  |  | 1 | | 2 | |
| No. | Statement | Q rank | z-score | Q rank | z-score |
| 1 | I use the tactile paving | -2 | -0.880^*^ | -1 | -0.414 |
| 2 | Cars always stop to let me cross the shared zone | -1 | -0.382^*^ | -5 | -1.638 |
| 3 | I know when a car is going to stop to let me cross the shared zone | 1 | 0.469* | -2 | -0.845 |
| 4 | Bollards get in my way^b^ | -3 | -1.088 | -3 | -1.075 |
| 5 | I use the beep at traffic lights to know when to cross the road^b^ | 0 | 0.349 | 1 | 0.378 |
| 6 | It is easy to see oncoming cars in a shared zone^b^ | 1 | 0.575 | 2 | 0.790 |
| 7 | The road and footpath should be separate | 0 | 0.342* | 4 | 1.437 |
| 8 | It is easy for drivers to see me in the shared zone | 1 | 0.529* | -1 | -0.266 |
| 9 | Drivers follow the road rules in a shared zone | 0 | 0.092* | -2 | -0.569 |
| 10 | More zebra crossings would make it easier for me | -2 | -0.419* | 1 | 0.425 |
| 11 | Cars feel too close to me at the zebra crossing | -3 | -1.310* | -1 | -0.161 |
| 12 | I know when to cross a shared zone^a^ | 3 | 1.009^^^ | 4 | 1.416 |
| 13 | Drivers make eye contact with me in the shared zone | -1 | -0.020* | -2 | -0.634 |
| 14 | I know where to cross a shared zone^a^ | 2 | 0.606^^^ | 3 | 0.949 |
| 15 | Signs help me to stay safe from cars^b^ | 0 | 0.162 | 0 | 0.105 |
| 16 | Shared zones are dangerous | -3 | -1.071* | 0 | 0.126 |
| 17 | Signs in the shared zone need to be bigger | -1 | -0.414* | 2 | 0.593 |
| 18 | I stay away from zebra crossings | -4 | -1.553* | -3 | -0.989 |
| 19 | I feel confident making eye contact with people^b^ | 2 | 0.632 | 2 | 0.882 |
| 20 | I feel secure being in a shared zone^b^ | 2 | 0.791 | 2 | 0.473 |
| 21 | I like it when there are lots of people around | -2 | -0.684* | -1 | -0.261 |
| 22 | I don’t like it when it is noisy | -1 | -0.168* | 1 | 0.388 |
| 23 | I feel confident crossing a shared zone^b^ | 5 | 1.348 | 3 | 1.119 |
| 24 | I feel confident crossing the road at a zebra crossing^b^ | 5 | 1.339 | 4 | 1.212 |
| 25 | It is important that I can walk by myself across a shared zone^b^ | 3 | 1.044 | 3 | 1.165 |
| 26 | I need someone with me when I cross a zebra crossing^b^ | -5 | -1.841 | -5 | -1.789 |
| 27 | The road rules at a zebra crossing are hard to understand | -4 | -1.474* | -2 | -0.481 |
| 28 | Drivers follow the rules of a zebra crossing | 0 | 0.221* | -4 | -1.456 |
| 29 | I stay away from shared zones^b^ | -4 | -1.357 | -4 | -1.267 |
| 30 | I know when a car is going to stop and let me cross a zebra crossing | 2 | 0.846* | -4 | -1.444 |
| 31 | It is important that I can walk by myself across a zebra crossing | 3 | 1.072* | 6 | 1.776 |
| 32 | Cars feel too close to me in the shared zone | -2 | -1.028* | 0 | -0.060 |
| 33 | Cars stop to let me cross the road at a zebra crossing | 1 | 0.510* | -3 | -0.865 |
| 34 | Drivers make eye contact with me at a zebra crossing | 0 | 0.054* | -3 | -1.251 |
| 35 | I feel confident crossing the road at traffic lights | 6 | 1.535* | 3 | 1.009 |
| 36 | It is easy to see oncoming cars at the zebra crossing | 3 | 0.970* | 0 | 0.154 |
| 37 | It is easy for drivers to see me at a zebra crossing | 1 | 0.453* | 0 | -0.156 |
| 38 | I feel secure using a zebra crossing | 4 | 1.253* | 1 | 0.250 |
| 39 | I know when to cross a zebra crossing^a^ | 4 | 1.114^^^ | 5 | 1.455 |
| 40 | I need someone with me when I am in a shared zone^b^ | -6 | -1.910 | -6 | -2.109 |
| 41 | I know where to cross the road at a zebra crossing^b^ | 4 | 1.299 | 5 | 1.489 |
| 42 | Zebra crossings are dangerous | -5 | -1.771* | 0 | -0.061 |
| 43 | More shared zones would make it easier for me^b^ | -1 | -0.195 | -1 | -0.246 |
| 44 | The road rules of the shared zone are hard to understand | -3 | -1.050* | 1 | 0.444 |
